# A multidecadal assessment of climate indices over Europe

**DOI:** 10.1038/s41597-020-0464-0

**Published:** 2020-04-28

**Authors:** Fernando Domínguez-Castro, Fergus Reig, Sergio M. Vicente-Serrano, Enric Aguilar, Dhais Peña-Angulo, Iván Noguera, Jesús Revuelto, Gerard van der Schrier, Ahmed M. El Kenawy

**Affiliations:** 1 ARAID Foundation, Zaragoza, 50018 Spain; 2 Departamento de Geografía y Ordenación del Territorio, Universidad de Zaragoza, Zaragoza, 50009 Spain; 3 Instituto Pirenaico de Ecología, Consejo Superior de Investigaciones Científicas (IPE–CSIC), Zaragoza, 50059 Spain; 4 Center for Climate Change, Universitat Rovira i Virgili, Tarragona, 43480 Spain; 5 Royal Netherlands Meteorological Institute (KNMI), 3730 AE De Bilt, Netherlands; 6 Department of Geography, Sultan Qaboos University, Al Khoud, Muscat, 123 Oman; 7 Department of Geography, Mansoura University, Mansoura, 35516 Egypt

**Keywords:** Climate change, Atmospheric science

## Abstract

Monitoring and management of several environmental and socioeconomic sectors require climate data that can be summarized using a set of standard and meaningful climate metrics. This study describes a newly developed gridded dataset for the whole of Europe, which employed a set of 125 climate indices spanning different periods based on data availability, but mainly 1950–2017 and 1979–2017. This dataset comprehensively summarizes climate variability in Europe for a wide range of climate variables and conditions, including air temperature, precipitation, biometeorology, aridity, continentality, drought, amongst others. Climate indices were computed at different temporal scales (i.e. monthly, seasonal and annual) and mapped at a grid interval of 0.25°. We intend to update these indices on an annual basis. This dataset is freely available to research and end-user communities.

## Abstract (summary)

**Table:** 

Measurement(s)	climate
Technology Type(s)	computational modeling technique
Factor Type(s)	air temperature • precipitation • sea level pressure • wind at 10 m • wind gust at 10 m • TOA radiation • radiation • insolation • snow density • snow depth • snowfall • total cloud cover • low cloud cover
Sample Characteristic - Environment	climate
Sample Characteristic - Location	Europe

Machine-accessible metadata file describing the reported data: 10.6084/m9.figshare.12029427

## Background & Summary

Climate processes are complex and therefore hard to monitor and quantify.This is mainly due to the different environmental and socioeconomic impacts of climate, which pose further challenges to climate quantification and assessment^1–3^. Although climate observations usually correspond to quantitative variables with a comprehensible physical meaning, the environmental, societal and economic impacts, including a number of sectors, usually depend on specific climate conditions or event characteristics^4–7^. For example, the cumulative climate conditions over long periods, the extreme values recorded over a period, the frequency of days with specific characteristics, the frequency of events above or below specific thresholds; amongst others, could have a direct influence on environmental and socioeconomic systems^8–11^. In the same context, monitoring and assessing the impact of climate is a complicated process, given that conditions that trigger specific impacts are often a result of interactions between different climate covariates. For example, bioclimatic conditions that strongly determine human health and comfort depend on a wide range of climate variables, such as air temperature, relative humidity, wind speed, etc.^12,13^. Similarly, while drought severity is mainly controlled by precipitation, other climate variables (e.g. atmospheric evaporative demand, potential evaporation) could contribute significantly to drought dynamics^14–16^.

Within this context, much effort has been made to develop synthetic climate indices, which can be employed to determine climate impacts on different natural systems and socioeconomic sectors^17–21^. These indices are also useful for providing a comprehensive assessment of climate variability and change processes under current and future climate change scenarios^22–25^. The joint Ccl/WCRP/JCOMM Expert Team on Climate Change Detection and Indices (ETCCDI)^26^ developed an array of standard climate indices, which have widely been used to determine recent trends in climate conditions, with a particular focus on precipitation and temperature metrics. These indices have been widely used over different world regions such as Brazil^27^, China^28,29^, Africa^30,31^, the Mediterranean^32,33^, Indonesia^34^, North America^35^, central America^36^, central Asia^37^, amongst others. In addition, these indices have also been analyzed to assess the consequences of projections for future climate change scenarios. For example, Aerenson *et al*.^38^ have recently analyzed a suite of climate indices based on daily precipitation and temperature projections at the global scale, with the aim of determining possible future changes under 1.5 °C and 2 °C warming scenarios. Their study indicated that temperature indices are likely to witness significant changes in the future. On the other hand, the behavior of precipitation indices is more complex and different scenarios may cause different changes in the intensity of precipitation. Other studies (e.g. Dong *et al*.^39^) employed climate indices to verify that the observed warming trends in Asia in the past six decades were inconsistent with the natural variability of the climate system, but agreed with climate responses to external forcing, as simulated by the models.

The availability of long-term, high spatial resolution and updated climate indices could be promising for the research community through a multidecadal assessment of climate change processes and their impacts. This climate information could also be useful for a wide variety of environmental and socioeconomic sectors (e.g. land and agricultural management, climate-based health impacts, insurance plans associated with weather extremes, etc). Currently, some climate indices datasets have been developed^35,40^, mostly with the purpose of analyzing trends in climate conditions, focusing more on extreme meteorological events^41,42^. There are some worldwide updated climate indices dataset, such as i) DegDays_0p25_1970_2018^43^, which provides indices summarizing monthly and annual cooling and heating degree –days at the global scale, ii) GHCNDEX^41^, which includes a wide range of the ETCCDI indices calculated at the global scale, and iii) the global drought monitoring dataset based on the standardized precipitation evapotranspiration index^44^. Nevertheless, in Europe, there is no updated dataset of a wide variety of climate indices that can be useful not only for climate analysis, but also for a broad sectorial impact assessment. For this reason, the objective of this study is to provide an updated gridded dataset of a large variety of climate indices that summarize the temporal and spatial variability of climate in Europe during the past seven decades (from 1950 to 2017)^45^. Understanding climate services as “*The transformation of climate-related data – together with other relevant information – into customized products such as projections, forecasts, information, trends, economic analyses, assessments (including technology assessments), counselling on best practices, development and evaluation of solutions and any other service in relation to climate that may be of use for the society at large”*^46^ makes this dataset a potential asset from a climate service perspective. The target beneficiaries of this dataset may include climate services providers, civil society groups, farmers, amongst different stakeholders and end-users.

## Methods

### Source datasets

To calculate the different climate indices, we employed two information sources: the European Climate Assessment & Dataset (ECA&D) E-OBS gridded dataset (https://www.ecad.eu/) and the ERA5 dataset (https://www.ecmwf.int/en/forecasts/datasets/reanalysis-datasets/era5). E-OBS dataset provides updated high quality daily air temperature, precipitation and sea level pressure data for Europe at different grid intervals from January 1950 onwards, with regular updates. This dataset was created from quality-controlled meteorological records^47^, sourced from the European National Meteorological Services. Earlier versions of this dataset have been made available at a spatial resolution of 0.25°, being improved (0.1°) in the version v17.0e. Herein, we produced our indices at a spatial resolution of 0.25° to be consistent with the resolution of ERA5. In pursuit of the ERA4CS INDECIS project (European Union Grant 690462), daily station data from ECA&D were homogenized^48^ and the adjusted records will serve as a basis for the upcoming version of E-OBS datasets. In our gridded dataset, the E-OBS dataset acted as the main base for calculating climate indices related to air temperature and precipitation.

ERA5 is the fifth generation of the re-analysis dataset developed by the European Centre for Medium-Range Weather Forecasts (ECMWF)^49^. Albeit with the availability of daily air temperature and precipitation data within ERA5, our preference was made to use ERA5 as a supplementary data source to the observational datasets, with the aim of securing unavailable observational data for some climate variables. This included data for dewpoint temperature at 2 m, wind at 10 m, wind gust at 10 m, top-of-atmosphere (TOA) forcing, radiation, insolation, snow density, snow depth, snowfall, total cloud cover, and low cloud cover. Data from ERA5 were provided at a spatial resolution of 0.25° and hourly frequency. However, we aggregated the hourly data to daily scale to match the temporal resolution of E-OBS. To date, ERA5 is only available from February 1979; however, we will utilize the dataset for the early decades (1950–1978) once data is available.

**Fig. 1:**
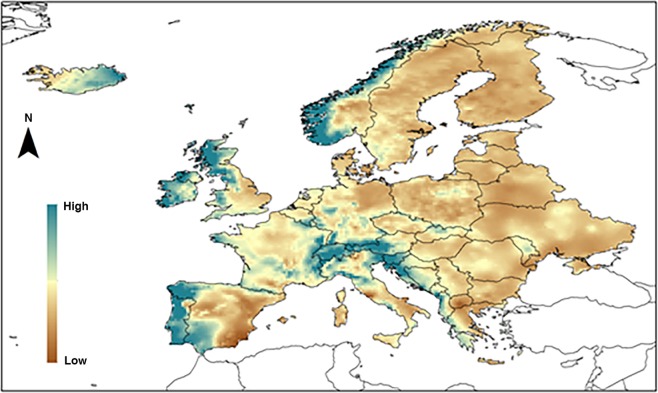
Spatial domain of the INDECISbase, the Modified Fournier Index [MFI] is represented.

Figure 1 depcits the spatial coverage of the study domain;our dataset included the entire European continent, apart from Russia, Turkey and Cyprus, comprising a total of 12280 series at a spatial resolution of 0.25°.

### Index calculation

We calculated a set of 125 climate indices, based on Climlnd: a recently developed package within R platform (https://cran.r-project.org/web/packages/ClimInd/index.html). The climate indices were grouped into eight broad categories: (i) temperature-based (N = 42), (ii) precipitation-based (N = 21), (iii) bioclimatic (N = 21), (iv) wind-based (N = 5), v) aridity/continentality (N = 10), vi) snow-based (N = 12), vii) cloud/radiation-based (N = 6), and (viii) drought (N = 8). Online-only Table 1 lists these indices and their description for the eight categories. The specific formulation of each index can also be consulted via: https://cran.r-project.org/web/packages/ClimInd/ClimInd.pdf. Overall, the majority of the indices were computed on monthly, seasonal (winter: DJF, spring: MAM, summer: JJA, autumn: SON) and annual scales. However, in some instances, specific indices were calculated only on the annual scale (e.g. the growing season precipitation [GSR] and the modified Fournier index [MFI]). For those indices that require a base period for their calculation (e.g. percentile-based indices), we considered the entire period as a reference period. Herein, the 125 climate indices were computed for each one of the 12280 series covering the European continent.

**Fig. 2:**
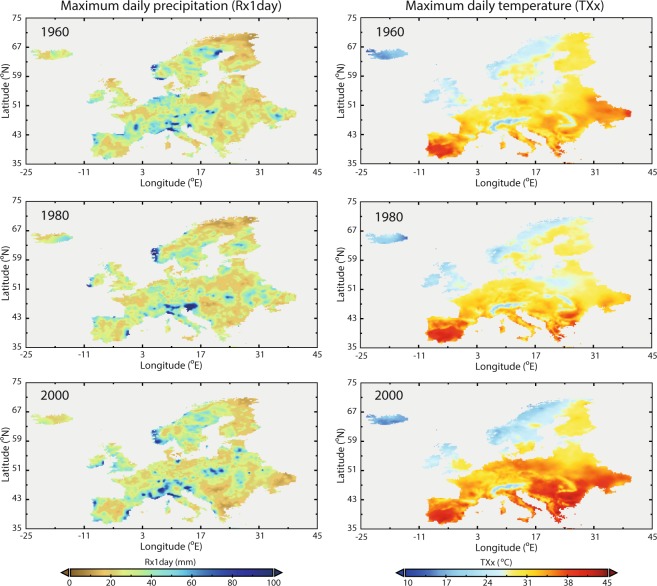
Spatial distribution of two selected climate indices (TXx and Rx1day) at the annual scale for three specific years (1960, 1980 and 2000).

Figure 2 illustrates the spatial variability of two selected indices (Rx1day and TXx) calculated on the annual scale for three specific years. Rx1day represents the maximum precipitation gauged within one day, whilst TXx refers to the highest daily maximum air temperature recorded during the year.

**Fig. 3:**
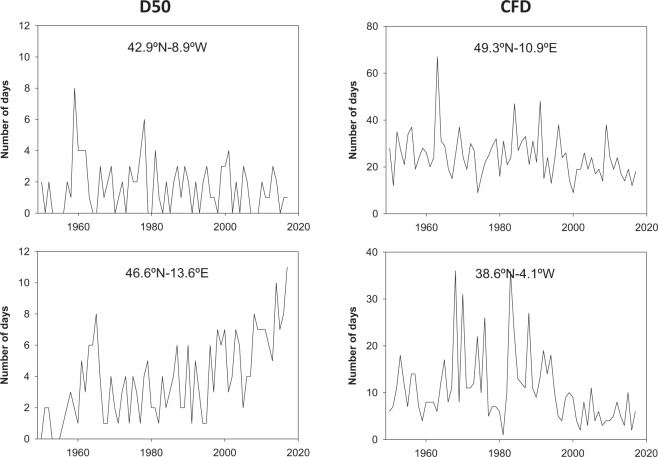
Temporal evolution of the annual D50mm and CFD indices calculated for specific grid points in Europe.

Figure 3 depicts the temporal evolution of two selected climate indices (heavy precipitation days [D50mm] and the maximum consecutive frost days [CFD]) for randomly selected grid points in Europe. The two indices were computed on the annual scale for the entire study period.

## Data Records

The climate index dataset has been archived in figshare (10.6084/m9.figshare.c.4773491)^45^, including all climate indices spanning the periods from 1950 or 1979 to 2017, based on data availability (see Online-only Table 1). Given the high number of indices included in this dataset, we have stored the climate index data in different levels. The first level corresponds to the main eight categories: temperature-based, precipitation-based, bioclimatic, wind-based, aridity/continentality, snow-based, cloud/radiation-based, and drought. Each of the first level categories (folders) is divided into subcategories (sub-folders); each of them corresponds to an individual climate index. Finally, at the third level, there are the data of each index at the different available temporal scales (i.e. monthly, seasonal and annual). The data were stored in a 3-D netcdf4 format (https://www.unidata.ucar.edu/software/netcdf/docs/netcdf_introduction.html). In specific, each file has an array of 464 (longitudes) × 201 (latitudes) × 68 (times for the annual resolution for the period 1950–2017). Indeed, the time dimension of the array varies as a function of the time resolution (i.e. monthly, seasonal, annual), as well as the period of time available (currently 1950 to 2017 for E-OBS indices and 1979 to 2017 for ERA5 indices). The geographical extent of the dataset is 25.37°N–75.37°N and 40.37°W–75.37°E. Netcdf4 files can be visualized and manipulated with several types of software, including open source software like R, Panoply, etc., and Geographic Information System software (ArcGIS©, QGIS). A complete list of the available software can be found at https://www.unidata.ucar.edu/software/netcdf/software.html. The entire dataset comprises almost 1 billion of data divided between the 125 indices and the different temporal scales (i.e. monthly, seasonal and annual).

## Technical Validation

The validation of our dataset depends largely on the reliability of climatic information used for calculating each index. The quality control and homogeneity of climatic data retrieved from the source datasets (i.e. E-OBS and ERA5) are comprehensively described via https://www.ecad.eu/download/ensembles/download.php for E-OBS and https://www.ecmwf.int/en/forecasts/datasets/reanalysis-datasets/era5 for ERA5. We have checked the spatial and temporal consistency of the obtained climate indices. Also, the monthly, seasonal and annual values of the different indices were visually inspected to detect any possible problems.

## Usage Notes

**Fig. 4:**
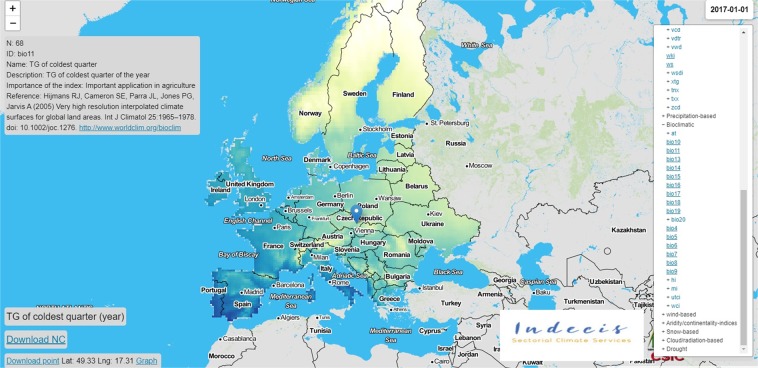
Web-tool to visualize the INDECIS dataset and download the entire dataset corresponding to any spatial or temporal query.

**Fig. 5:**
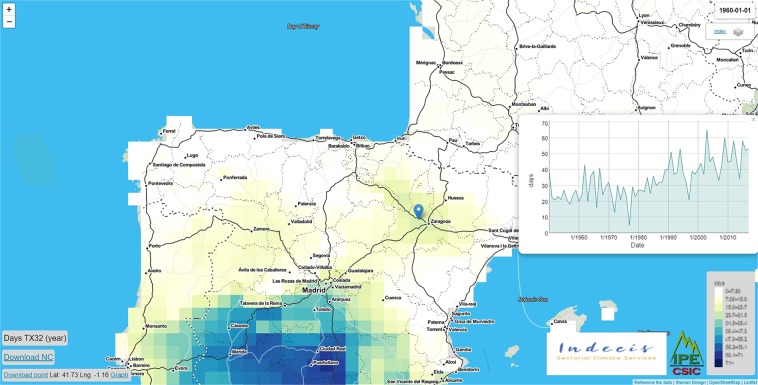
A representative example showing the temporal variability of a selected index (D32: annual temperature sums for days with mean temperatures above 32 °C) for a specific grid point.

In addition to the stored dataset in the figshare repository, the climate indices are made also available through the project website (https://indecis.csic.es/), maintained by the Spanish National Research Council (Fig. 4). In addition to its role as a data warehouse, the project’s website can be used as a climate service, where end-user beneficiaries can select, visualize and download any index of interest for any grid point in Europe (Fig. 5). These series can be downloaded in a generic ASCII .txt format. The web site also allows for the navigation between the monthly, seasonal and annual archives and directly download any netcdf4 of the database. The entire dataset stored in this web site will be updated regularly depending on the availability and updates of the ECA&D and ERA5 data. This website is mirrored at the portal of the INDECIS project (http://www.indecis.eu/indices.php).

## Data Availability

The code used to calculate the indices is available via: https://cran.r-project.org/web/packages/ClimInd/index.html. The R scripts necessary to calculate the different indices, from the ECA&D and ERA5 gridded datasets in a 3-D array format, can be accessed via: https://gitlab.com/fergusrg/indecis_example.

## Online-only Table

**Online-only Table 1:** A list of the climate indices included in the INDECIS database and their main characteristics.

Category	Name	Acronym	Units	Data provider	Starting date
Temperature-based	Mean of daily maximum air temperature	GTX	°C	E-OBS	1950
	**Maximum of daily maximum air temperature**	TXx	°C	E-OBS	1950
	**Minimum of daily maximum air temperature**	TXn	°C	E-OBS	1950
	Mean of daily minimum air temperature	GTN	°C	E-OBS	1950
	**Maximum of daily minimum air temperature**	TNx	°C	E-OBS	1950
	**Minimum of daily minimum air temperature**	TNn	°C	E-OBS	1950
	Mean of daily mean air temperature	GTG	°C	E-OBS	1950
	Maximum of daily mean air temperature	XTG	°C	E-OBS	1950
	Minimum of daily mean air temperature	NTG	°C	E-OBS	1950
	**Cold days**	TX10p	%	E-OBS	1950
	**Cold nights**	TN10p	%	E-OBS	1950
	**Cold spell duration**	CSDI	Days	E-OBS	1950
	**Diurnal temperature range**	DTR	°C	E-OBS	1950
	Mean daily difference of diurnal temperature range	vDTR	°C	E-OBS	1950
	**Frost days**	FD	Days	E-OBS	1950
	**Growing season length**	GSL	Days	E-OBS	1950
	**Ice days**	ID	Days	E-OBS	1950
	Maximum consecutive frost days	CFD	Days	E-OBS	1950
	Extreme temperature range	ETR	°C	E-OBS	1950
	**Summer days**	SU	Days	E-OBS	1950
	Maximum consecutive summer days	CSD	Days	E-OBS	1950
	Difference days above/below maximum temperature 17	DD17	Days	E-OBS	1950
	**Tropical nights**	TR	Days	E-OBS	1950
	Heating degree days	HD17	°C	E-OBS	1950
	Very cold days	VCD	Days	E-OBS	1950
	Very warm days	VWD	Days	E-OBS	1950
	**Warm days**	TX90p	Days	E-OBS	1950
	**Warm nights**	TN90p	Days	E-OBS	1950
	**Warm spell duration**	WSDI	Days	E-OBS	1950
	Zero crossing days	ZCD	Days	E-OBS	1950
	Onset of growing season 6 days	OGS6	date	E-OBS	1950
	Onset of growing season 10 days	OGS10	date	E-OBS	1950
	Growing season (Apr-Oct)	Ta_o	°C	E-OBS	1950
	Growing season(May-Sep)	Tm_s	°C	E-OBS	1950
	Growing degree days	GD4	°C	E-OBS	1950
	Winkler index	WKI	°C	E-OBS	1950
	Winter Severity	WS	°C	E-OBS	1950
	Sum of degree days when maximum temperature > = 32	STX32	°C	E-OBS	1950
	Number of days with maximum temperature > = 32	D32	Days	E-OBS	1950
	Sum of degree days when minimum temperature < = −15	STN15	°C	E-OBS	1950
	Sum of degree days when minimum temperature < = −10	STN10	°C	E-OBS	1950
	Sums positive	PTG	°C	E-OBS	1950
Precipitation-based	Total precipitation	Rt	mm	E-OBS	1950
	**Maximun precipitation**	RX1day	mm	E-OBS	1950
	**Days precipitation ≥10 mm**	R10mm	Days	E-OBS	1950
	**Days precipitation ≥20 mm**	R20mm	Days	E-OBS	1950
	**Maximum consecutive 5-day precipitation**	Rx5day	mm	E-OBS	1950
	**Simple daily intensity index**	SDII	mm/Day	E-OBS	1950
	Dry days	DD	Days	E-OBS	1950
	Effective precipitation	EP	mm	E-OBS	1950
	**Longest dry period**	CDD	Days	E-OBS	1950
	**Longest wet period**	CWD	Days	E-OBS	1950
	**Precipitation fraction very wet days**	R95tot	%	E-OBS	1950
	**Precipitation fraction extremely wet days**	R99tot	%	E-OBS	1950
	Heavy precipitation days	D50mm	Days	E-OBS	1950
	Very wet days	D95p	Days	E-OBS	1950
	Precipitation Concentration Index	PCI	Dimensional values	E-OBS	1950
	Modified Fournier Index	MFI	Dimensional values	E-OBS	1950
	Growing season precipitation	GSR	mm	E-OBS	1950
	Non-growing season precipitation	NGSR	mm	E-OBS	1950
	**Total precipitation wet days**	PRCPTOT	mm	E-OBS	1950
	Wet days 1 mm	DR1mm	Days	E-OBS	1950
	Wet days 3 mm	DR3mm	Days	E-OBS	1950
Bioclimatic	Mean temperature warmest quarter	BIO10	°C	E-OBS	1950
	Mean temperature coldest quarter	BIO11	°C	E-OBS	1950
	Precipitation wettest month	BIO13	mm	E-OBS	1950
	Precipitation driest month	BIO14	mm	E-OBS	1950
	Coefficient of variation precipitation	BIO15	%	E-OBS	1950
	Precipitation wettest quarter	BIO16	mm	E-OBS	1950
	Precipitation driest quarter	BIO17	mm	E-OBS	1950
	Precipitation warmest quarter	BIO18	mm	E-OBS	1950
	Precipitation coldest quarter	BIO19	mm	E-OBS	1950
	Temperature seasonality	BIO4	°C	E-OBS	1950
	Maximum temperature warmest month	BIO5	°C	E-OBS	1950
	Minimum temperature coldest month	BIO6	°C	E-OBS	1950
	Difference warmest/coldest month	BIO7	°C	E-OBS	1950
	Mean temperature of the wettest quarter	BIO8	°C	E-OBS	1950
	Mean temperature of the driest quarter	BIO9	°C	E-OBS	1950
	Mean radiation	BIO20	W m-2	ERA5	1979
	Universal thermal climate index	UTCI	°C	ERA5	1979
	Mould index	MI	Days	ERA5	1979
	Heat index	HI	°F	E-OBS	1950
	Wind chill index	WCI	°C	ERA5	1979
	Apparent temperature	AT	°C	ERA5	1979
wind-based	Days wind gusts above 21 m/s	DFx21	Days	ERA5	1979
	Daily maximum wind gust	FXx	m/s	ERA5	1979
	Mean of daily mean wind strength	FG	m/s	ERA5	1979
	Calm days	fgcalm	Days	ERA5	1979
	Days daily averaged wind above 10.8 m/s	FG6Bft	Days	ERA5	1979
aridity/continentality-indices	Reference evapotranspiration	ETo	mm	ERA5	1979
	UNEP aridity index	UAI	index	ERA5	1979
	Climatic moisture deficit	CMD	mm	ERA5	1979
	De Martonne aridity index	MAI	Dimensional values	E-OBS	1950
	Emberger aridity index	EAI	Dimensional values	E-OBS	1950
	Johansson Continentality Index	JCI	Dimensional values	E-OBS	1950
	Kerner Oceanity Index	KOI	Dimensional values	E-OBS	1950
	Pinna Combinative index	PiCI	Dimensional values	E-OBS	1950
	Budyko Index	BI	Dimensional values	ERA5	1979
	Marsz Oceanity Index	MOI	Dimensional values	E-OBS	1950
snow-based	Snowfall sum	SS	mm	ERA5	1979
	Snow days depth 1–10 cm	SD0_10	Days	ERA5	1979
	Snow days depth 10–20 cm	SD10_20	Days	ERA5	1979
	Frequency of snow days	FSD	Dayss	ERA5	1979
	Mild snowy days	MSD	Dayss	ERA5	1979
	Heavy snowy days	HSD	Dayss	ERA5	1979
	Date of first snow cover	FSC	Date	ERA5	1979
	Date of first permanent snow cover	FPSC	Date	ERA5	1979
	Date of last permanent snow cover	LPSC	Date	ERA5	1979
	Average snow depth	ASD	m	ERA5	1979
	Amount of snow covered days	SCD	Days	ERA5	1979
	Maximum snow depth	MS	m	ERA5	1979
Cloud/radiation	Sum of sunshine duration	SSD	h	ERA5	1979
	Sunny days	SND	Days	ERA5	1979
	Foggy days	FOD	Days	ERA5	1979
	Mean daily cloud cover	CC	%	ERA5	1979
	Sunshine duration fraction	SSp	Dimensional values	ERA5	1979
	Atmospheric Clarity Index	ACI	%	ERA5	1979
Drought	Standardized precipitation index 1	SPI1	z units	ERA5	1979
	Standardized precipitation index 3	SPI3	z units	ERA5	1979
	Standardized precipitation index 6	SPI6	z units	ERA5	1979
	Standardized precipitation index 12	SPI12	z units	ERA5	1979
	Standardised Precipitation-Evapotranspiration Index 1	SPEI1	z units	ERA5	1979
	Standardised Precipitation-Evapotranspiration Index 3	SPEI3	z units	ERA5	1979
	Standardised Precipitation-Evapotranspiration Index 6	SPEI6	z units	ERA5	1979
	Standardised Precipitation-Evapotranspiration Index 12	SPEI12	z units	ERA5	1979

The indices given in bold refer to the ETCCDI indices.
